# Distribution and Evolution of Repeated Sequences in Genomes of Triatominae (Hemiptera-Reduviidae) Inferred from Genomic In Situ Hybridization

**DOI:** 10.1371/journal.pone.0114298

**Published:** 2014-12-05

**Authors:** Sebastian Pita, Francisco Panzera, Antonio Sánchez, Yanina Panzera, Teresa Palomeque, Pedro Lorite

**Affiliations:** 1 Sección Genética Evolutiva, Facultad de Ciencias, Universidad de la República, Montevideo, Uruguay; 2 Departamento de Biología Experimental, Área de Genética, Universidad de Jaén, Jaén, Spain; Universidade Federal do Rio de Janeiro, Brazil

## Abstract

The subfamily Triatominae, vectors of Chagas disease, comprises 140 species characterized by a highly homogeneous chromosome number. We analyzed the chromosomal distribution and evolution of repeated sequences in Triatominae genomes by Genomic in situ Hybridization using *Triatoma delpontei* and *Triatoma infestans* genomic DNAs as probes. Hybridizations were performed on their own chromosomes and on nine species included in six genera from the two main tribes: Triatomini and Rhodniini. Genomic probes clearly generate two different hybridization patterns, dispersed or accumulated in specific regions or chromosomes. The three used probes generate the same hybridization pattern in each species. However, these patterns are species-specific. In closely related species, the probes strongly hybridized in the autosomal heterochromatic regions, resembling C-banding and DAPI patterns. However, in more distant species these co-localizations are not observed. The heterochromatic Y chromosome is constituted by highly repeated sequences, which is conserved among 10 species of Triatomini tribe suggesting be an ancestral character for this group. However, the Y chromosome in Rhodniini tribe is markedly different, supporting the early evolutionary dichotomy between both tribes. In some species, sex chromosomes and autosomes shared repeated sequences, suggesting meiotic chromatin exchanges among these heterologous chromosomes. Our GISH analyses enabled us to acquire not only reliable information about autosomal repeated sequences distribution but also an insight into sex chromosome evolution in Triatominae. Furthermore, the differentiation obtained by GISH might be a valuable marker to establish phylogenetic relationships and to test the controversial origin of the Triatominae subfamily.

## Introduction

The repetitive DNAs distribution along chromosomes is one of the essential elements in evolutionary genetics for understanding the organization and the evolution of genomes [1]. Analyses of these sequences are even more important in organisms with holocentric chromosomes, such as hemipteran insects, where the lack of primary constriction, small chromosome size and the limited banding procedures makes chromosomal studies harder to achieve.

The subfamily Triatominae include 140 species called kissing bugs, vectors of Chagas disease or American trypanosomiasis, recognized as the most serious human parasitic disease of Latin America with around 7–8 million people infected [2]. Karyotypic information is currently available for more than 80 species, showing a highly conserved diploid chromosome number, ranging from 21 to 25 chromosomes in males [3]. The number of autosomes is remarkably constant; all species except 3 present 20 autosomes. They have three sex systems in males (XY, X_1_X_2_Y and X_1_X_2_X_3_Y), being the sex chromosomes achiasmatic and showing a particular segregation called inverted meiosis or post-reduccional segregation [4].

In triatomines, the heterochromatin (revealed by C-banding) and the 45S ribosomal genes chromosomal mapping (revealed by fluorescent in situ hybridization) are the principal repetitive DNAs used for studying karyotypic diversification and make evolutionary inferences [3], [5], [6]. Heterochromatin variation includes remarkable changes in the quantity, size, composition, chromosome location and behavior of autosomal C-blocks during cell divisions. Autosomal C-heterochromatin differences are positively correlated with an extensive variation in the total DNA content measured by laser flow cytometry. The haploid genome size varies 4-fold, from 0.72 pg in *Rhodnius* species to 2.90 pg in *Triatoma delpontei* [3], [7]. FISH technique application in 46 triatomine species has also shown a high variability in the 45S ribosomal DNA chromosomal location, never reported so far in holocentric chromosomes, demonstrating that these repeated DNA sequences are an important marker to disclose chromosomal differentiation [5], [6].

The most striking example of intraspecific variation of repeated sequences is *Triatoma infestans*, which involved polymorphism in the number of heterochromatic chromosomes, different molecular composition within the C-blocks and variation on the rDNA genes localization [5], [8]–[11]. This species comprises two main evolutionary lineages, known as the Andean and non-Andean groups, defined by substantial differences (from 30% to 50%) in nuclear DNA amount, due to dissimilar quantities of highly repeated DNA revealed as C-heterochromatin [8]. In Andean group, the number of autosomes with heterochromatin fluctuates from 14 to 20, while in the non-Andean group varies from 4 to 7 [8]. Fluorochromes staining shown that the C-blocks in both chromosomal groups are conformed by different DNA repeats [10], [11]. In the three largest autosomal pairs, each C-block is subdivided into two different repeats regions: a telomeric DAPI-positive region (AT-rich) and a subtelomeric chromomycin A3-positive region (GC-rich). In the other autosomes, the C-blocks are exclusively formed by a DAPI positive telomeric region. The Y chromosome is almost totally C-heterochromatic and DAPI positive, while that the X chromosome is polymorphic. In non-Andean group this chromosome is euchromatic with a DAPI-positive signal in one chromosomal end [10], [12] while that in Andean group, the X chromosome is almost entirely C-heterochromatic and displayed two DAPI bands on each chromosomal end [13].

Inter-species distribution of repeated sequences by Genomic in situ hybridization (GISH) was applied in different organisms with dissimilar objectives such as genome analysis, determination of phylogenetic relationships, detection of chromosomal aberrations and alien chromatin [14]. In holocentric systems, GISH approaches have been applied primarily to the study of the evolution of sex chromosomes, particularly in Lepidoptera [15]. In Heteroptera, only one report has been published to explore the evolution of neo-sex chromosomes in the genus *Dysdercus* (Pyrrhocoridae) [16].

The aim of this paper is to analyze the similarities and differences of repeated DNA sequences among several Triatominae species by means of GISH. For this purpose, we made three genomic probes derived from the total DNA of two triatomine species with the highest DNA content of the subfamily: *T. delpontei* and *T. infestans* (Andean Group) [7]. These DNA probes were hybridized both on their own chromosomes (self-GISH) and on other species included in six different genera, belonging to the two main Triatominae tribes. With this strategy, we can establish a preliminary but broad overview on the repeated sequences evolution both in autosomes and sex chromosomes in the subfamily Triatominae.

## Materials and Methods

### Material

Table 1 summarizes the geographic origin, relevant cytogenetic traits and GISH results of the material here analyzed. No specific permissions were required for insect collections performed in this work, and did not involve endangered or protected species.

**Table 1:** Geographic origin, relevant cytogenetic traits and GISH results of the eleven Triatominae species analyzed by three genomic probes.

SPECIES	Male Diploid Number (2n)	Amount (%) autosomal C-heterochromatin	Chromosome location of Autosomal C-bands	X chromosome	GISH results. Hybridization signals	Geographic Origin. Country: Department, Locality, habitat
*TRIBE TRIATOMINI*						
*Triatoma delpontei*	20A + XY	Polymorphic, 45–50%	9–10 autosomal pairs with large C-blocks in only one chromosomal end (Figure 1g)	Almost entirely C-heterochromatic	All chromatin. 9–10 bivalents and X chromosome with strongest hybridization signals in only one chromosomal end. Y chromosome almost totally labeled (Figure 1c and 1f)	Bolivia: Santa Cruz, Tita, S. 18° 34′ 31″ S, 62° 40′ 05″ W.
*Triatoma infestans Andean Group*	20A + XY	Polymorphic, 40–50%	7–9 autosomal pairs with C-blocks of different size in one or two chromosomal ends (Figure 1i)	Almost entirely C-heterochromatic	All chromatin. 6 bivalents with strongest hybridization signals (different size and chromosome location). Y chromosome intensively and totally labeled while that X chromosome has a small signal (Figure 1h)	Bolivia: Potosí, Palquiza, S. 21° 31′ 41″ S, 65° 45′ 04″ W, and Potosí, Thago Thago, S. 18° 00′ 44″ S, 65° 48′ 31″ W.
*Triatoma infestans Non-Andean Group*	20A + XY	Polymorphic, 24–30%	3 autosomal pairs with C-blocks in one or two chromosomal ends (Figures 2c and 2e)	Euchromatic	All chromatin. Hybridization signals strongest in 3 larger autosomal pairs and the whole Y chromosome. The X no have strong labeled (Figures 2b and 2d)	Argentina: Chaco. Tres Estacas, P. 26° 54′ 30″ S, 51° 40′ 23″ W.
*Triatoma platensis*	20A + XY	Polymorphic, 10–12%	2–4 autosomal pairs with small C-blocks in one or two chromosomal ends	Almost entirely C-heterochromatic	All chromatin. 3 largest bivalents with strong and small signals. Y and X chromosomes intensively and totally labeled (Figure 2f)	Uruguay: Paysandú, S. 32° 18′ 28″, 58° 02′ 59″ W.
*Mepraia spinolai*	20A + X_1_X_2_Y	Polymorphic, 15–25%	All autosomes with C-dots in one or two chromosomal ends (Figure 2l)	Small C-dots in both Xs chromosomes	All chromatin (Figures 2j and 2k). All bivalents with strong hybridization signals in chromosomal ends. Y chromosome intensively and totally labeled (Figure 2i)	Chile: Metropolitan Region of Santiago, Colina, S. 33° 11′ 53″ S, 70° 39′ 42″ W.
*Triatoma dimidiata*	20A + X_1_X_2_Y	Polymorphic, 5–10%	All autosomes with C-dots in one or both ends	Euchromatic	All chromatin. Only Y chromosome intensively and totally labeled (Figure 3b)	Guatemala: Jutiapa, Carrizal, D. 14° 25′ 48″ N, 89° 57′ 28″W
*Triatoma carrioni*	20A + XY	5%	2 autosomal pairs with C-dots in one chromosomal end	Euchromatic	All chromatin. Only Y chromosome intensively and totally labeled (Figure 3c)	Peru: Piura, Ayabaca, S. 4° 35′ 00″ S, 79° 43′ 00″ W.
*Triatoma protracta*	20A + X_1_X_2_Y	Polymorphic, 35–45%	All autosomes with C-blocks in one or two chromosomal ends (Figure 3e)	X_1_ with C-dots in both ends. X_2_ euchromatic	All chromatin. Only Y chromosome intensively and totally labeled (Figure 3d)	Insectary Justin Schmidt (USA). Origin colony: USA, Arizona, S.
*Dipetalogaster maxima*	20A + XY	0%	Without autosomal C-heterochromatin	Euchromatic	All chromatin. Only Y chromosome intensively and totally labeled (Figure 3f)	Mexico: Baja California Sur, La Paz, S. 24° 09′ N, 110° 17′ W.
*Eratyrus mucronatus*	20A + X_1_X_2_Y	Polymorphic, 0–5%	0–1 autosomal pair with C-blocks	Euchromatic	All chromatin. Only Y chromosome intensively and totally labeled (Figure 3g)	Insectary E. Chagas. Origin: Brazil, Para, S.
*Panstrongylus geniculatus*	20A + X_1_X_2_Y	0%	Without autosomal C-heterochromatin	Both X chromosomes with C-dots	All chromatin. Only Y chromosome intensively and totally labeled (Figure 3h)	Colombia: Antioquia, Amalfi, S. 6° 55′ 58″ N, 75° 05′ 30″ S.
*TRIBE RHODNIINI*						
*Rhodnius prolixus*	20A + XY	0%	Without autosomal C-heterochromatin	Euchromatic	Hybridization signals scattered throughout all chromosomes. No chromosomal region was observed with specific labeling, including the heterochromatic Y chromosome (Figure 3i)	Insectary CDC (USA). Origin: Colombia. S.

These genomic probes produce the same hybridization pattern in each species but the chromosomal location of the most intense signals allows recognizing a species-specific hybridization patterns. P =  peridomiciliary; D =  domiciliary; S =  sylvatic.

Chromosome preparations for GISH analyses were obtained from males of 11 triatomine species, included in the two principal tribes of the subfamily: Rhodniini and Triatomini (Table 1), which involve almost 90% of the 140 recognized species [17]. For Rhodniini tribe, which included 19 species in 2 genera, we analyzed one species: *Rhodnius prolixus*. For Triatomini tribe, which involved 104 species in eight genera, we studied species from the following five genera: *Dipetalogaster*, *Eratyrus*, *Mepraia*, *Panstrongylus* and *Triatoma*. For the *Triatoma* genus, we studied the three main clades or groups: a) the Rubrofasciata Group (from Central and North America and Old World species): *T. protracta* and *T. dimidiata*, b) the Dispar Group (west of the Amazon region): *Triatoma carrioni* and c) the Infestans Group (from south and east of the Amazon region). For this group we included three closely related species of the infestans subcomplex (*T. delpontei*, *T. infestans* and *T. platensis*). In *T. infestans* we analyzed the two chromosomal groups: Andean and non-Andean with striking differences in the amount and chromosome distribution of the C-heterochromatin, both in autosomes and sex chromosomes (see Table 1).

### Chromosome preparations and C-banding

For cytological preparations, testes were removed from adult insects alive, fixed in an ethanol–glacial acetic acid mixture (3∶1) and stored at -20°C. Squashes were made in a 50% acetic acid drop, coverslips were removed after freezing in liquid nitrogen and the slides were air dried and then stored at 4°C. C-banding was performed according to Panzera *et al.* [8]. The analysis of C-banded preparations was made using a Nikon Eclipse 80i microscope with a DS-5Mc-U2 digital camera.

### Genomic DNA Isolation and probe labeling for GISH techniques

Three genomic DNA probes were used from two species. Each one isolated from one adult individual: one from a male of *T. infestans* (Andean group) collected in Bolivia (Potosí, Palquiza, sylvatic) and the others two probes were obtained from one male and one female of *T. delpontei* collected from Bolivia (Santa Cruz, Tita, sylvatic). These two species have the highest DNA content in triatomines: *T. delpontei* (2.90 pg) and *T. infestans* -Andean group- (1.98 pg) [3]. In both species, C-heterochromatin amount represent approximately 45% of the autosomal complement [18], but with different chromosome localization (see Table 1).

Genomic DNA was purified from adult legs following the NucleoSpin Tissue kit (MACHEREY-NAGEL). For the probes, total genomic DNAs were labeled with biotin-16-dUTP (Roche) using a Nick Translation Kit (Roche), following manufacturer's instructions.

In situ hybridization was carried out as described previously [19]. Hybridization solutions were prepared to a final concentration of 0.5–2 ng probe/mL in 50% formamide. Hybridization was conducted at 37°C overnight. Fluorescence immunological detection was performed using the avidin-FICT/anti-avidin-biotin system with two amplification rounds. Slides were mounted with Vectashield (Vector). DAPI in the antifade solution was used to counterstain chromosomes. The hybridized chromosomes were observed and photographed using a BX51 Olympus fluorescence microscope equipped with a CCD camera (Olympus DP70) and merged using the DPManager software. Hybridization pattern for each species was determined by the chromosomal analyses of at least two individuals.

## Results

**Figure 1:**
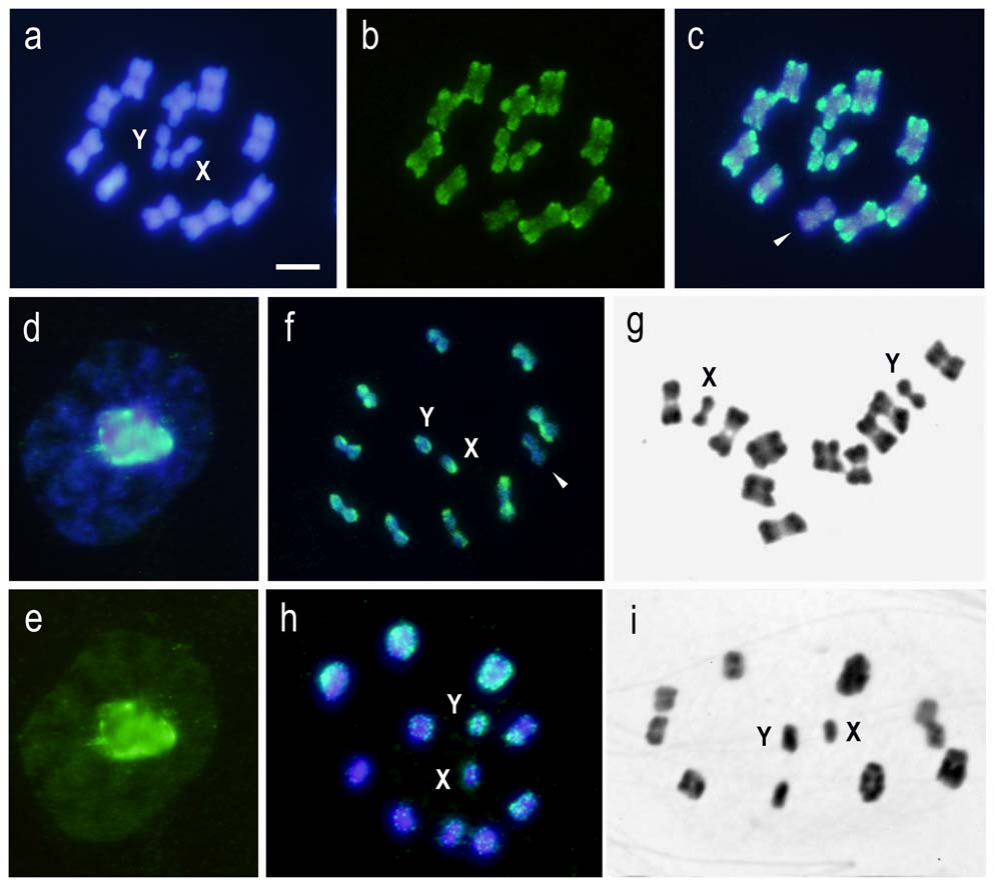
Self-GISH using three genomic DNA (gDNA) probes: *Triatoma delpontei* (TD) (male or female) and *Triatoma infestans* (TI) (Andean group) on own chromosomes. Bar  = 5 µm. (a-b-c) TD gDNA on TD chromosomes. Metaphase I (2n = 20A + XY). **(a)** DAPI staining. **(b)** FITC. **(c)** Merged. Hybridization signals appear scattered on all chromatin. Furthermore, strongest signals are preferably located at one chromosomal end on nine of the ten autosomal bivalents. Both sex chromosomes (X and Y) appear almost entirely labeled. The bivalent without signal is pointed out by arrowhead. **(d–e)** TD gDNA on TD. Early male meiotic prophase. **(d)** DAPI staining. **(e)** FITC. A large heteropycnotic chromocenter appear with a strong hybridization signal and the rest of the chromatin with weaker signals. **(f)** TD gDNA (female) on TD chromosomes. Metaphase II (2n = 20A + XY). Terminal regions of 9 autosomal pairs and both sex chromosomes, including the Y chromosome, appear strongly labeled. The autosomal pair without strong hybridization signals is pointed out by arrowhead. **(g)** TD. C-banding. Metaphase I. All autosomal bivalents (10) show C-blocks in only one chromosomal end. Both sex chromosomes (XY) appear almost entirely. C-heterochromatin distribution similar as observed with GISH in (c). **(h)** TI (Andean group) gDNA on TI (Andean group) chromosomes. Metaphase I (2n = 20A + XY). Six autosomal bivalents show hybridization signals with different intensity and size. The Y chromosome appears almost entirely labeled while that X chromosome shows a small hybridization region. **(i)** TI (Andean group). C-banding. Metaphase I. Seven to nine autosomal bivalents appear heterochromatic with C-blocks of different size in one or both chromosomal ends, while X and Y sex chromosomes are almost entirely C-heterochromatic.

**Figure 2:**
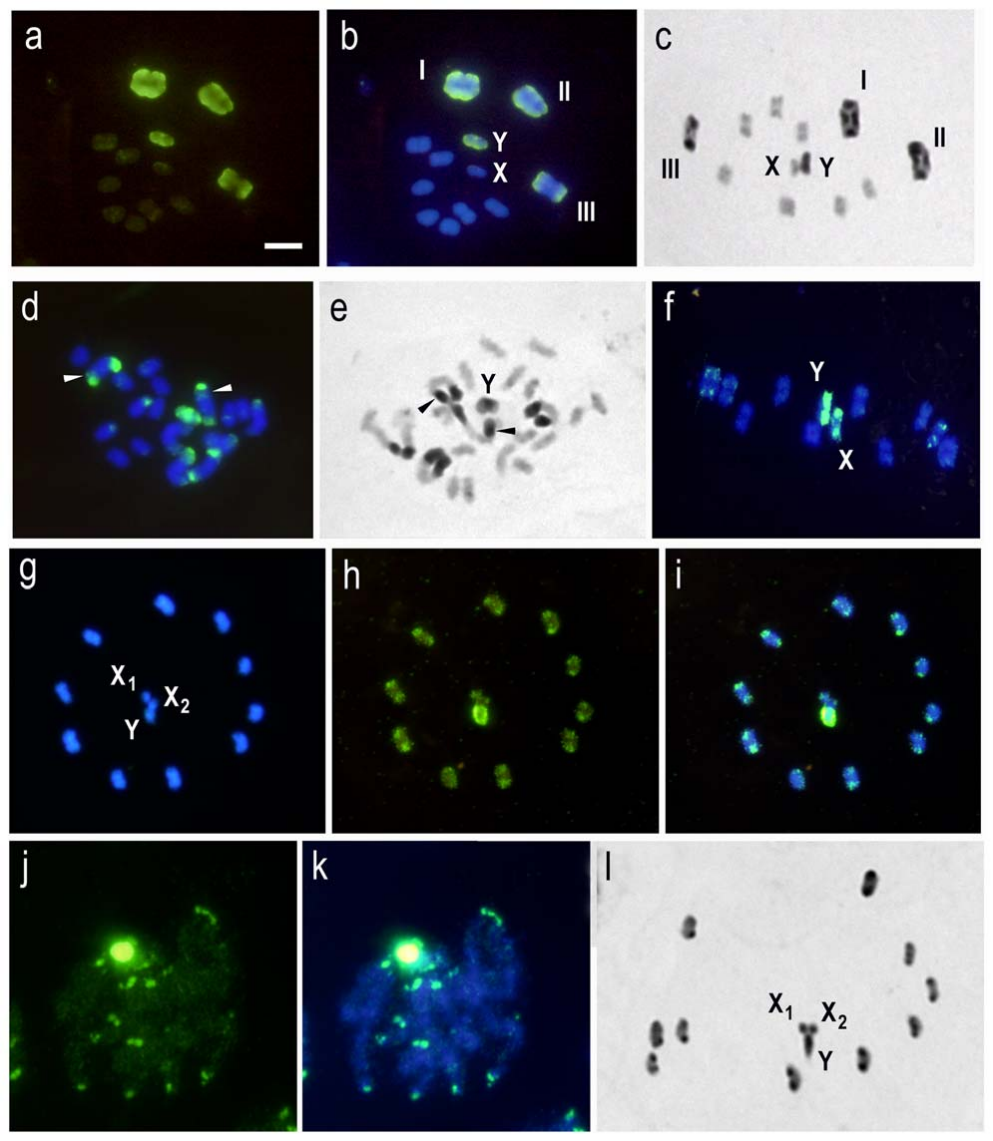
Inter-specific GISH of triatomine species using three genomic DNA (gDNA) probes: *Triatoma delpontei* (TD) (male or female) and *Triatoma infestans* (TI) (Andean group). Bar  = 5 µm. (a–b) TI (Andean group) gDNA on TI (non-Andean group) chromosomes. Metaphase I (2n = 20A + XY). **(a)** FITC. **(b)** Merged. All chromatin appear labeled. Strong hybridization signals are restricted to the three largest autosomal pairs and the Y chromosome: in two largest bivalents (I and II) the signals are localized in both chromosomal ends while that in the third bivalent (III) the label in restricted to only one chromosomal end. The remaining seven autosomal bivalents and X chromosome did not display strong labeling. **(c)** TI (non-Andean group). C-banding. Metaphase I. Three bivalents present C-blocks: two of them in both chromosomal ends (I and II) and the third in only one end (III). The Y chromosome is C-positive, and seven autosomal bivalents and X chromosome are euchromatic. C-heterochromatin distribution similar as observed with GISH in (b). **(d)** TD gDNA on TI (non-Andean group) chromosomes. Spermatogonial mitotic prometaphase (2n = 22 chromosomes). Six autosomes appear with strong and telomeric hybridization signals: 4 of them in both chromosomal ends and the other 2 in only one telomeric region. The Y chromosome is entirely hybridized. Arrowheads pointed out subtelomeric regions (DAPI negative) without GISH label localized on the largest autosomes. **(e)** C-banding. TI (non-Andean group). Spermatogonial mitotic prometaphase (2n = 22). Six autosomes with C-blocks: 4 of them in both chromosomal ends, and the other 2 in only one end. The Y chromosome appears almost totally C-heterochromatic. Each autosomal C-block is subdivided into 2 regions: a darker subtelomeric region (arrowheads) and a clearer telomeric region. **(f)** TI (Andean group) gDNA on *T. platensis* chromosomes. Metaphase I (2n = 20A + XY). Hybridization signals are restricted to small regions of 3 autosomal bivalents. Both sex chromosomes (X and Y) appear almost entirely labeled. **(g-h-i)** TI (Andean group) gDNA on *M. spinolai* chromosomes. Metaphase II (2n = 20A + X_1_X_2_Y). **(g)** DAPI staining. **(h)** FITC. **(i)** Merged. All chromatin appear labeled. Autosomal telomeric regions present small and intense hybridization signals. The Y chromosome appears strongly and totally labeled. **(j–k)** TI (Andean group) gDNA on *Mepraia spinolai* chromosomes. Early male meiotic prophase. **(j)** FITC **(k)** Merged. All chromatin appear labeled but strong signals are restricted to telomeric regions and on the meiotic chromocenter constituted by the association of sex chromosomes with some autosomes. **(l)** *M. spinolai*. C-banding. Metaphase II (2n = 20A + X_1_X_2_Y). C-heterochromatin distribution similar as observed with GISH in **(c)**.

**Figure 3:**
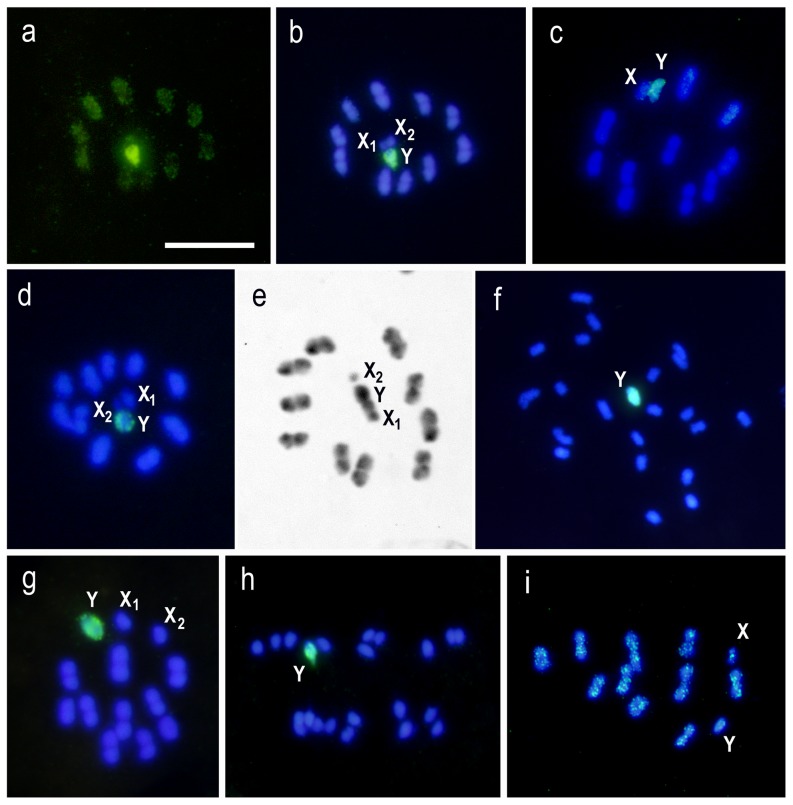
Inter-specific GISH of triatomine species using three genomic DNA (gDNA) probes: *Triatoma delpontei* (TD) (male or female) and *Triatoma infestans* (TI) (Andean group). Bar  = 5 µm. (a–b) TD gDNA on *Triatoma dimidiata* chromosomes. Metaphase II (2n = 20A + X_1_X_2_Y). **(a)** FITC **(b)** Merged. All chromatin present dispersal hybridization signals but only the Y chromosome exhibits a very strong labeled. (c) TI (Andean group) gDNA on *T. carrioni* chromosomes. Metaphase I (2n = 20A + XY). Strong hybridization signals are restricted to the heterochromatic Y chromosome. **(d)** TD gDNA on *T. protracta* chromosomes. Metaphase II (2n = 20A + X_1_X_2_Y). Only the Y chromosome presents strong hybridization signal in spite of the autosomal C-bands in all autosomal pairs and in one X chromosome. **(e)** *T. protracta*. C-banding. Metaphase II. All autosomal pairs, the Y chromosome and one X chromosome present C-blocks in one or both chromosomal ends. **(f)** TD gDNA on *Dipetalogaster maxima* chromosomes. Spermatogonial mitotic prometaphase with 22 chromosomes (2n = 20A + XY). Only the Y chromosome presents intense hybridization signal. **(g)** TI (Andean group) gDNA on *Eratyrus mucronatus* chromosomes. Metaphase I (2n = 20A + X_1_X_2_Y). Only the Y chromosome presents hybridization signal. **(h)** TI (Andean group) gDNA on *Panstrongylus geniculatus* chromosomes. Anaphase II (2n = 20A + X_1_X_2_Y) showing a post-reductional sex chromosomes segregation. Only the Y chromosome has strong hybridization signals. **(i)** TI (Andean group) gDNA on *Rhodnius prolixus* chromosomes. Metaphase I (2n = 20A + XY). The hybridization signals are scattered throughout all chromosomes. No chromosomal region was observed with intense labeled signals, including the heterochromatic Y chromosome.

Our GISH results reveal the occurrence of two chromosomal hybridization configurations: a) very intense hybridization signals concentrated on specific chromosomal regions or particular chromosomes and b) lower intensity hybridization signals dispersed along all chromosomes (Figures 1, 2 and 3). In some cases the lower intense hybridization signals dispersed along the chromosomes were masked by the DAPI signal in the merged figures. The three genomic probes produce the same hybridization pattern in each species. The chromosomal location of the most intense signals allows recognize a species-specific hybridization patterns.

### Self-GISH or Auto-GISH. Genomic DNA from *T. delpontei* (male or female) on *T. delpontei* male meiotic chromosomes

All chromatin present scattered hybridization signals. In addition 9 of the 10 bivalents and the X chromosome exhibit strongest hybridization signals in only one chromosomal end, while the Y chromosome appears almost totally hybridized (Figures 1a to 1c). This *T. delpontei* individual does not have C-block in one autosomal bivalent (data not shown), consequently this chromosome do no have hybridization signals on the chromosomal ends (Figures 1b and 1c). Male meiotic prophase clearly shows the two hybridization configurations: a large heteropycnotic chromocenter with a strong hybridization signal and the rest of the chromatin with weaker signals (Figures 1d and 1e). This meiotic chromocenter is formed by the heterologous association of the C-heteropycnotic regions of all autosomal bivalents and both sex chromosomes [18]. In metaphase II, each autosomal pair has a strong signal in only one chromosomal end and both sex chromosomes (X and Y) appear labeled (Figure 1f). This GISH pattern is very similar compared with those observed with C-banding: figure 1g shows a C-banded metaphase I with a C-block in only one chromosomal end of all bivalents while both sex chromosomes are C-heterochromatic (compare Figure 1c and 1g).

### Self-GISH or Auto-GISH. Genomic DNA from *T. infestans* Andean Group (male) on *T. infestans* Andean Group male meiotic chromosomes

All chromosomes show hybridization signals but with differences in size and intensity. The strongest signals are observed in the terminal regions of 6 bivalents, in the whole Y chromosome and more scarcely on the X chromosome (Figure 1h). Also in this case the hybridization pattern is very similar to the C-banding pattern: *T. infestans* (Andean group) exhibit 7–9 autosomal bivalents with C-blocks of different size in one or both chromosomal ends. Both sex chromosomes (X and Y) appear C-heterochromatic (Figure 1i).

### Inter-specific GISH

Genomic probes produce a specific pattern on each one of the 11 species analyzed. However, since each probe recognizes the same repeated sequences, the chromosome hybridizations results on each species are the same with the three applied probes. For this reason, to illustrate the patterns of the 11 analyzed species we combine different genomic probes in figures 1, 2 and 3. Genomic DNA probe of *T. infestans* (Andean group) on *T. delpontei* chromosomes and the reverse hybridization, i.e., *T. delpontei* gDNA probe on *T. infestans* (Andean group) chromosomes shows the same patterns described in the corresponding self-GISH.

Genomic DNA probe of *T. infestans* (Andean group) on *T. infestans* (non-Andean group) chromosomes shown strong hybridization signals on the Y chromosome (almost entirely) and on the three largest autosomal bivalents (Figures 2a and 2b). These signals are localized in both chromosomal ends of the two largest bivalents (I and II, Figure 2b) and in one end of the third largest autosomal pair (III, Figure 2b). The remaining seven autosomal bivalents and X chromosome not display strong labeling. Similar pattern is observed with C-banding (Figure 2c). On the mitotic chromosomes, more detail about the chromosomal localization of the hybridization signals can be seen (Figure 2d). The Y chromosome appears almost completely labeled. Terminal or telomeric positions of the autosomal hybridization signals are clearly observed. Furthermore a subterminal region (negative for GISH and DAPI) is observed between the telomeric hybridization signal and the euchromatin (Figure 2d, arrowheads). This pattern is similar to C-banding, in which the Y chromosome and six autosomes (three pairs) appear with C-blocks in one or both chromosomal ends (Figure 2e). The size of the C-heterochromatic regions is larger than those obtained with GISH, and they are formed by a darker subtelomeric region (arrowheads) and a clearer telomeric region with Giemsa staining. These results indicated that telomeric C-bands are strongly hybridized with the GISH probes while the subtelomeric regions are not.

In *T. platensis*, the three largest autosomal bivalents present small but intense signals on only one chromosomal end and both sex chromosomes (X and Y) are almost entirely hybridized (Figure 2f). This GISH pattern is very similar to the described by C-banding (Table 1). In *M. spinolai*, all chromatin appear labeled but the ten autosomal pairs exhibit strong hybridization dots on chromosomal ends, clearly observed during early meiotic prophase and metaphase II (Figures 2g to 2k). The Y chromosome appears strongly and totally labeled (Figures 2h to 2k). This hybridization pattern is similar to C-banding pattern (Figure 2l). In the other seven Triatomine tribe species, including *Triatoma*, *Dipetalogaster*, *Eratyrus*, and *Panstrongylus* genera, strong hybridization signal is only observed on the C-heterochromatic Y chromosome (Figures 3a to 3d, and 3f to 3h). However, some of these species exhibit autosomal C-heterochromatin (Table 1), which clearly is not labeled with our GISH probes, as observed in autosomes and one X chromosome of *T. protracta* (compare Figures 3d with 3e).

Finally in *R. prolixus* from the Rhodniini tribe, a species with C-band only on the Y chromosome, the hybridization signals are scattered throughout all chromosomes (Figure 3i). No chromosomal region was observed with specific labeling, including the heterochromatic Y chromosome.

## Discussion

Heteropteran have holocentric or holokinetic chromosomes, i.e. chromosomes with diffuse or non-localized centromeres [4]. The absence of a primary constriction, the similar and small chromosome size and the minor number of chromosomal landmarks limited comparative and evolutionary chromosomal studies in holocentric systems. In many different heteropteran groups, including triatomines, the main source of karyological differentiation has been the identification of highly repeated DNA regions included in the heterochromatin, revealed by the classical C-banding technique [3], [20]. Fluorescent banding and FISH with ribosomal DNA probes (18S and 28S rDNA) have shown a high chromosomal diversity in triatomines [5], [6], [11]. Unlike other insects such as Orthoptera, Diptera and Coleoptera, FISH analyses with DNA probes of other repeated sequences, such as the 5S rDNA cluster and histone genes [21], failed to achieve satisfactory results in Heteroptera. To better understand the chromatin organization and composition of the holocentric chromosomes it is essential to find methodological approaches that allow the detection of other repeated sequences.

Our GISH results reveal the occurrence of two hybridization configurations: scattered hybridization signals along the entire chromosome length and more intense hybridization signals concentrated in specific chromosomal regions or the whole chromosomes (Figures 1, 2 and 3). Most likely both hybridization types are reflecting two main classes of repeated DNA elements within eukaryotic genomes: dispersed and accumulated repeats. Regions with accumulated repeats, usually C-band positive, comprise mainly satellite DNAs (included in the heterochromatin) and multigene families such ribosomal RNA (rRNA) and the histone gene families [22], although dispersed repeats could be also present in euchromatic regions [23], [24]. The dispersed repeats mainly include transposable genetic elements. In several insect species, such as *Drosophila* and *Anopheles*, transposable elements constitute about 15% of its genome [25]. The scatter hybridization configuration here obtained is a typical pattern observed in other insects with probes of different mobile genetic elements [26].

All analyzed species in this paper exhibit scattered hybridizations signals on their chromatin, suggesting that transposable elements constituted a very important component in the triatomine genomes. Furthermore, all species except *R. prolixus*, present a specific intense hybridization pattern on regions of autosomes and/or sex chromosomes that vary in number, size, and chromosomal localization. *R. prolixus* present all chromatin, including Y chromosome, with dispersed hybridization without strong hybridization regions, suggesting that their repetitive DNA is mainly formed by dispersed repeats without cluster repeats, at least recognized by our genomic probes. Hence, the heterochromatic Y chromosome in *R. prolixus* is constituted by different repetitive sequences than the observed in Triatomini species (Figure 3i). Previous studies suggest the existence of different families of transposable elements in this species [27], [28].

In summary, these results reveal that the genomes of the 10 tribe Triatomini studied species share dispersed repetitive DNA sequences. In addition *T. infestans*, *T. delpontei, T. platensis* and *Mepraia spinolai* share also the accumulated repetitive sequences located in the C-band positive regions, while in the remaining species these sequences are only present on the heterochromatic Y chromosome. Among Triatomini species and *Rhodnius prolixus* (Tribe Rhodniini) only dispersed repetitive DNA sequences are shared.

### Correlation between GISH and DAPI positive C-band

Genome-specific repeats have frequently a non-random distribution, forming clusters within heterochromatin blocks. In most organisms, including many plant species, the GISH hybridization signals often coincide with C-bands and also with DAPI positive regions, suggesting the presence of AT-rich DNA sequences at these regions [14]. Our GISH results in triatomines are partially consistent with that observed in plants, when evolutionarily related species are compared. However, the correspondence between C and DAPI patterns with GISH hybridizations are not observed when more evolutionarily distant species are analyzed.

In the three closely related infestans subcomplex species (*T. infestans, T. delpontei*, and *T. platensis*) and *Mepraia spinolai*, the strong hybridization signals mainly co-localized with their C-banding patterns (Figures 1, 2 and 3). However, more detailed analysis in *T. infestans* demonstrated that GISH signals coincide exactly with autosomal DAPI positive regions described for this species (Figure 2d). Hence, our hybridization signals are not able to label the subterminal CMA_3_ positive C-heterochromatic regions (GC-rich) observed in the largest autosomal pairs of *T. infestans* (arrowheads Figures 2d and 2e). This concordance between GISH patterns and DAPI regions in the infestans subcomplex and *M. spinolai* species is not observed when we analyze more evolutionarily distant species, even within the genus *Triatoma*. For example, the DAPI positive C-heterochromatin regions in all autosomes and X chromosome of *T. protracta* do not show strong hybridization signals (compare Figures 3d and 3e). Similar results were obtained in other species with autosomal C-heterochromatin such as *T. dimidiata* and *T. carrioni* (Figures 3b and 3c). Our GISH results are in agreement with the Triatominae molecular phylogeny, which shown that *M. spinolai* is closed relater with South American than North American *Triatoma* species [29]. Hence, repetitive sequences shared between *M. spinolai* and infestans subcomplex species is showing this evolutionary relationship.

Recent analyses of the AT-rich satDNA portion of *T. infestans* using reassociation kinetics (C_0_t) found two repetitive arrays located on the terminal regions of autosomal C-heterochromatin but not on the sex chromosomes heterochromatin [13]. Due to the similar chromosome localization, our GISH probes probably identify these repetitive arrays (Figure 2d) and others A–T rich sequences present on the C-heterochromatin of the X and Y sex chromosomes of *T. infestans*, not identified by C_0_t studies (Figures 1h, 2b and 2d).

These results clearly show that: (a) infestans subcomplex closely related species share the majority of their repetitive sequences, in spite of their different size and chromosome distribution, (b) our genomic probes identify different repetitive DNA families, which are A-T rich, (c) autosomal and X heterochromatin regions from other evolutionary distant species are integrated by other divergent repetitive DNA, without homology to our genomics probes, hence not identified by GISH. Isolation and characterization of repeated sequences, either by GISH or others methodological approaches, seems to be appropriate genetic markers to infer evolutionary relationships between different species groups.

### Evolution of sex chromosomes inferred by GISH

In all triatomine species, sex chromosomes are very well differentiated from autosomes by their particular behavior during meiosis. They are considered asynaptic and achiasmatic during male meiotic division [30], [31] showing an inverted meiosis: in first meiotic division the sister chromatids of each sex chromosome separate equationally and in the second division the sex chromosomes segregate to opposite poles [4].

In insect groups such as Diptera, Orthoptera and Lepidoptera distinct DNAs classes have been mapped on sex chromosomes [32]–[35]. However in Heteroptera there is a lack of knowledge about the molecular composition and evolution of sex chromosomes. In all of the 80 cytogenetically Triatominae studied species, the Y chromosome is almost entirely C-heterochromatic [3]. Molecular studies on the Y chromosome sequences are limited to fluorescence banding, showing that it is constituted by DAPI positive AT-rich sequences [10], [12], [13]. Our GISH studies reveal that in the ten Triatomini species, the Y chromosome is mainly constituted by accumulated repetitive sequences. It is well known that most Y chromosomes (from mammals, fish, insects and plants) are enriched with repeats in a process of heterochromatin accumulation. However, a striking result is that 10 species of triatomines, including 5 different genera, present and share the same types of repeated DNA sequences. In Diptera and Lepidoptera, unlike triatomines, Y chromosome (or their equivalent W chromosome) sequence composition appears to have a rather high turnover evolution rate because even in related species, the Y chromosome exhibit different repeated sequences or gene content [34], [36]. For this reason the Y chromosome repeated sequences conservation in Triatomini tribe species here analyzed is a very uncommon phenomenon and probably these repeated sequences represent an ancestral character of this tribe (Figures 1, 2 and 3).

On the other hand, the *R. prolixus* Y chromosome, species which belongs to the Rhodniini tribe, does not shared the same conserved repetitive DNA sequences observed in the analyzed Triatomini species (Figure 3i). This result suggests an early evolutionary differentiation or dichotomy within Triatominae subfamily in two clearly distinct clades: the Rhodniini and Triatomini tribes. Our results are in agreement with phylogenetic studies with nuclear and mitochondrial DNA sequences which suggested a Rhodniini–Triatomini node as the oldest known split within the triatomine bugs [39], [40]. Another explanation could be a different origin of the Triatomini and Rhodniini Y chromosomes, either deriving from different autosomal pairs or B chromosomes as suggested in homopteran and *Drosophila* species [41].

In triatomines, sex chromosomes are achiasmatic, so surely completely differentiated between each other, which implicate a scenario with an old Y chromosome. As has been described in *Drosophila* and mammals, old Y chromosome evolution is mostly driven by genetic hitchhiking, where evidence of positive selection evidence was found [36], [37]. In addition, the Y chromosome is a very important determinant of male fitness [38], so conservation in triatomines could be caused by a selective pressure on important male fitness genes. Maybe genome project data on Y-linked genes would bring light on this question.

One of the most striking results of this paper is that, in infestans subcomplex and *M. spinolai* species, the sex chromosomes (Xs and Y) with each other and with some autosomes share highly repeated sequences (Figures 1 and 2). A distinctive cytogenetic feature of these species is that during meiotic prophase chromosomal associations among autosomes with both sex chromosomes occur [18], [42]. These associations would facilitate the sequences exchange among these non-homologous chromosomes [9]. If we consider that the highly repeated sequences localized in the Y chromosome are the plesiomorphic state, a possible scenario could be a sequences transfer from Y chromosome to the autosomes and X chromosomes. Consequently, the sequence homology between them could be considered a secondary character (apomorphic) restricted to closely related species groups. Comparative analyses on the 45S rDNA clusters chromosome position in several triatomine species also suggested the existence of chromatin exchange between sex chromosomes [6], in contrast to the widely accepted idea that the achiasmatic sex chromosomes of Heteroptera do not interchange sequences [31]. Furthermore, the simultaneous presence of 45S ribosomal clusters in autosomes and the X chromosome observed in *T. infestans* and *T. delpontei* also suggests the occurrence of sequences exchanges among autosomes and sex chromosomes [5], [9]. It has been suggested that transposable elements could play an important role in the mobilization of repeat sequences among chromosomes [23]. Nevertheless, in order to advance in the knowledge of sex chromosomes evolution, a molecular characterization of their sequences will be necessary.

### Phylogenetic origin of the subfamily Triatominae

The phylogenetic origin of blood-feeding Triatominae has received considerable attention due to the epidemiological significance as vectors of Chagas disease. Conflicting hypotheses support Triatominae as monophyletic [29], [39], [43], polyphyletic [17], [44] or paraphyletic group [40]. The answer to this question goes beyond the academic interest, since it could represent a very important issue to know if hematophagy in Triatominae arose from a single evolutionary event or as multiple independent evolutionary processes. According to the polyphyletic and paraphyletic proposals, the Triatomini and Rhodniini tribes are derived from quite different reduviid subfamilies. The sister group of the Triatomini seems most likely to be the Reduviinae, while the sister group for the Rhodniini may possibly be the Stenopodainae or Salyavatinae [17], [43]. Considering the extreme Y chromosome molecular differentiation between the two tribes described here, the application of GISH methodology on the suspected sister species of Triatominae could help elucidate the monophyletic or polyphyletic origin of this subfamily.
